# Warming and drought combine to increase pest insect fitness on urban trees

**DOI:** 10.1371/journal.pone.0173844

**Published:** 2017-03-09

**Authors:** Adam G. Dale, Steven D. Frank

**Affiliations:** 1 Entomology and Nematology Department, University of Florida, Gainesville, Florida, United States of America; 2 Department of Entomology and Plant Pathology, North Carolina State University, Raleigh, North Carolina, United States of America; Natural Resources Canada, CANADA

## Abstract

Urban habitats are characterized by impervious surfaces, which increase temperatures and reduce water availability to plants. The effects of these conditions on herbivorous insects are not well understood, but may provide insight into future conditions. Three primary hypotheses have been proposed to explain why multiple herbivorous arthropods are more abundant and damaging in cities, and support has been found for each. First, less complex vegetation may reduce biological control of pests. Second, plant stress can increase plant quality for pests. And third, urban warming can directly increase pest fitness and abundance. These hypotheses are not mutually exclusive, and the effects of temperature and plant stress are particularly related. Thus, we test the hypothesis that urban warming and drought stress combine to increase the fitness and abundance of the scale insect, *Melanaspis tenebricosa*, an urban tree pest that is more abundant in urban than rural areas of the southeastern U.S. We did this by manipulating drought stress across an existing mosaic of urban warming. We found support for the additive effect of temperature and drought stress such that female embryo production and body size increased with temperature and was greater on drought-stressed than watered trees. This study provides further evidence that drivers of pest insect outbreaks act in concert, rather than independently, and calls for more research that manipulates multiple abiotic factors related to urbanization and climate change to predict their effects on ecological interactions. As cities expand and the climate changes, warmer temperatures and drought conditions may become more widespread in the native range of this pest. These changes have direct physiological benefits for *M*. *tenebricosa*, and potentially other pests, that may increase their fitness and abundance in urban and natural forests.

## Introduction

Urban forests are often more heavily infested with herbivorous arthropod pests than surrounding rural areas [1]. There are multiple potential drivers of this, which are not well understood. One well supported hypothesis suggests that features of urban landscapes support fewer natural enemies, creating enemy-free space that allows herbivores to proliferate [2–4]. More recently, urban warming has been shown to play a major role and may outweigh factors associated with conservation biological control [5–7]. Temperature directly affects arthropod metabolism and development, which under warming may lead to larger, more fecund individuals [8–10]. Dale and Frank (2014b) found that 2°C of urban warming increased the abundance of *Melanaspis tenebricosa* (Hemiptera: Diaspididae), a scale insect pest, on *Acer rubrum* street trees. One mechanism for this was that *M*. *tenebricosa* on trees in warmer areas were larger and produced up to three times as many offspring as those on trees in cooler areas.

Urban warming, or the urban heat island effect, is driven by impervious surface cover, building density, and other anthropogenic factors [11]. Impervious surface cover around trees reduces water infiltration and availability to plant roots [12, 13]. At the same time, warming can induce drought stress by increasing the atmospheric demand for water [14, 15]. The plant stress hypothesis proposes that drought can increase herbivore fitness and abundance by increasing the nutritional quality of plants and reducing plant defenses [16, 17]. Sap-feeding pests in particular, often show a positive response to drought stress [18–20]. Several studies link the plant stress hypothesis to urbanization by showing positive relationships between impervious surface cover and herbivore abundance, or between drought, impervious surface, and herbivores [21–24]. For example, Cregg and Dix (2001) found that oak trees growing in downtown sidewalk tree pits were more drought-stressed and harbored more aphids and lace bugs than oaks planted in a nearby campus park with little impervious surface. Likewise, the density of *Pulvinaria regalis* on urban mulberry trees significantly increased as surrounding impervious surface cover increased [22].

Climate change research suggests that abiotic factors like temperature, CO_2_, and drought often act in concert or antagonistically to affect herbivores, which makes single-factor empirical tests less meaningful [25]. Given the close association between urban warming, impervious surface cover, and drought stress, it is difficult to separate their effects in observational experiments. Our overriding goal was to determine how drought stress and warming interact to affect the fecundity and population growth of *M*. *tenebricosa*, a sap feeding pest of urban trees in the southeastern U.S. Our first objective was to manipulate water stress in *A*. *rubrum* street trees across an existing gradient of urban temperatures to decouple these effects on *M*. *tenebricosa*. Our second objective was to determine the effect of drought stress and temperature on *M*. *tenebricosa* fitness by quantifying body size, embryo production, and abundance over three generations. Understanding the effects of warming and drought on urban plants and animals is important because urban forests support much of urban biodiversity and provide ecosystem services like cooling, air filtration, and aesthetic enhancement that benefit human and environmental health [26–28].

## Materials and methods

### Study organisms

The gloomy scale, *M*. *tenebricosa*, is a native armored scale insect pest of *Acer spp*. trees, primarily *A*. *rubrum*, which is the most common genus of landscape tree in the eastern U.S. [29–31]. *Acer rubrum* is native throughout eastern North America and comprises over 18% of the trees in Raleigh’s urban street tree inventory and approximately 15% of trees in natural forests in North Carolina’s piedmont region [32, 33]. *Melanaspis tenebricosa* are drastically more abundant on *A*. *rubrum* in urban than natural forests throughout the southeastern U.S. [29, 30, 34]. These insects feed on parenchyma cells within the trunk and branches of trees [35]. Heavy infestations cause trees to lose branches, prematurely drop leaves, and often die [29].

*Melanaspis tenebricosa* undergoes one generation per year, although they develop from nymph to adult within approximately five months (May-September) [36]. These insects spend the winter as adult females, then give live birth over 6 to 8 weeks beginning in May [29, 36]. Newly emerged nymphs are mobile for a few hours before settling on the bark, where they become sessile, produce a waxy cover, and feed for the remainder of their life. Nymphs become adults by approximately September, mate during the fall, and begin egg production soon after [36].

### Study system

**Fig 1:**
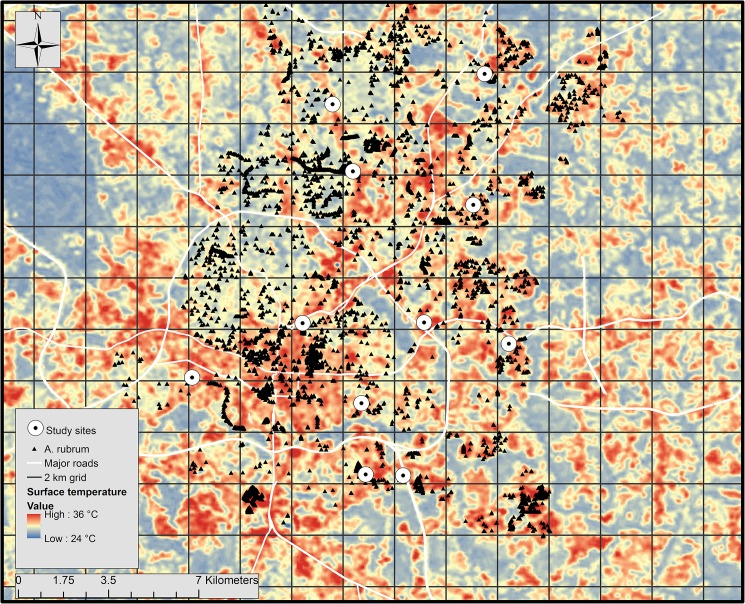
Map of *A*. *rubrum* street trees and study sites overlaid onto a thermal map of surface temperature in Raleigh, NC. Grid is made of 2x2 km squares.

This study was conducted in the city of Raleigh, North Carolina in the piedmont region of the southeastern U.S. Raleigh, NC has a temperate climate with an average annual precipitation and temperature of 117 cm and 15°C, respectively [37]. All trees used in the study were property of the City of Raleigh, NC, USA and located in the right-of-way, which was up to 10 m from the road’s edge. We were granted permission to conduct research on these trees by the Raleigh Parks, Recreation, and Cultural Resources Department. To select study sites, we used ArcMap 10.2 to overlay a geocoded street tree inventory map created by the city of Raleigh from 2010 to 2013 onto a Landsat thermal image of surface temperature acquired on 18 August 2007 and prepared as described in Meineke et al. [7]. Using random selection procedures as in Youngsteadt et al. [38], we created a grid of 2x2 km squares, divided the city into equal quadrants, and randomly selected three grid squares per quadrant (Fig 1). From each selected grid square, we picked a pair of *A*. *rubrum* from the hottest site and a pair from the coldest site based on Landsat surface temperature, resulting in 48 trees at 24 randomly selected sites. Warmest and coolest sites were selected within each randomly selected grid square to capture the heterogeneity of temperature in Raleigh’s urban forest and simplify the random selection procedure. Tree pairs at each site were between 15–75 m apart from one another following the selection procedure. Rather than using 24 sites as in Dale [39], we used 11 sites due to the logistical constraints of scale insects having to be measured and dissected within a short time after collecting from sites throughout the city. We selected the 11 sites because they represented trees across the gradient of site temperatures and all trees had some level of insect infestation from which we could sample. Tree diameter at breast height (DBH) ranged from 15.7 to 40.4 cm. Tree pairs at each site differed in DBH from 0.5 to 9.3 cm with an average (±SEM) of 3 (±1.2) cm.

### Effect of temperature and water treatment on drought stress

To determine tree canopy temperature, we placed an iButton thermochron DS1921G (Dallas Semiconductor, Dallas, TX, USA) remote temperature logger 4.5 m above ground within each tree’s canopy. iButtons were mounted on the underside of a lateral branch attached to an iButton wall mount within a 59-ml portion container (Dart Container Corporation, Mason, Michigan, USA) using a cable tie. iButtons recorded temperature every two hours from May 2014 through August 2014, the time period in which *M*. *tenebricosa* are most active and developing. Mean two-hour tree canopy temperature, henceforth referred to as tree canopy temperature, was calculated for the period May 2014 through August 2014 and used for all analyses of temperature.

One of our objectives is to determine the effect of drought stress. To create a difference in drought stress between trees at each site, one tree was watered and one tree was not. Eleven trees ranging from 15.7 to 40.4 cm in diameter at breast height received no supplemental water beyond natural precipitation. Ten trees with diameters of 15.7 to 31.8 cm were irrigated with two TreeGator ® slow-release watering bags (Spectrum Products Inc., Youngsville, NC) around their base. One tree was 37.7 cm in diameter and required three watering bags around its base for irrigation. Each bag held approximately 75 liters and was filled twice per week from May through August 2014 and 2015, resulting in 300 liters of water per tree per week and 450 liters per week for one tree.

To determine how temperature and watering treatments affected tree drought stress, we used a pressure chamber (PMS Instrument Company, Albany, OR) to measure midday xylem water potential of each tree once during the second week of June, July, and August 2014. Midday water potential was measured during 1100 to 1500 each day. We removed one 15 to 20 cm terminal twig from sun-exposed locations approximately 5.5 m above ground on the north and south-facing sides of each tree. Weather conditions during each measuring period were mostly sunny and between 29 and 31°C. Three-month mean xylem water potential was calculated for each tree and used for analysis.

### Effect of temperature and drought stress on *M*. *tenebricosa* fitness and abundance

To determine if *M*. *tenebricosa* were affected by temperature and drought stress, we measured adult female body size and embryo production, and calculated the ratio of third-generation adult females (counted September 2015) to first generation adult females (counted April 2014) on each tree. To determine the effect of temperature and drought stress on *M*. *tenebricosa* fitness, we measured and dissected adult female *M*. *tenebricosa* under a dissecting microscope and counted the number of embryos developing within them immediately prior to birth as in Dale & Frank (2014b). At the end of reproductive development, on 6 May 2015, we collected one, 30.5 cm twig from each cardinal direction and selected an adult female every 15 cm beginning at the terminal end, totaling twelve individuals per tree. Using the same technique as Dale and Frank [5], we measured adult female *M*. *tenebricosa* body length from the pygidium to the anterior end of the body. Then, we dissected each *M*. *tenebricosa* under a stereomicroscope, slide mounted the contents, and counted the developing embryos under a phase-contrast compound light microscope.

Finally, we determined live adult female *M*. *tenebricosa* abundance on each *A*. *rubrum* by pruning one 0.3 m live terminal twig, 5 m above ground, from each cardinal direction of the tree canopy as in Dale and Frank [40]. *Melanaspis tenebricosa* were collected and counted under a dissecting microscope at the beginning of the experiment in April 2014 and again in September 2015. Although our first collection was in 2014, it was of 2013-generation adult females that had overwintered and not yet reproduced. Our final collection in September 2015 was of 2015-generation adult females prior to overwintering, which allowed us to capture three generations of *M*. *tenebricosa* on each study tree. As a measure of overall population growth on each tree, we calculated the ratio of September 2015 to April 2014 adult females. Population growth ratio data were log transformed to increase the normality of residuals. During the summer of 2015, two study trees were cut down for utility clearance and landscape renovation. Therefore, *M*. *tenebricosa* abundance from September 2015 as well as population growth ratio data include 20 trees instead of 22.

### Statistical analyses

We quantified body size, embryo production, initial abundance, final abundance, and population growth ratios. Each of these measured dependent variables were analyzed separately using analysis of covariance (ANCOVA), with watering treatment as a categorical predictor and tree canopy temperature as a covariate. Analyses were conducted using PROC GLM and LSMEANS in SAS 9.4 (SAS Version 9.4, Cary, NC). Each ANCOVA was first run as the full model with each predictor (watering treatment, temperature) and the interaction term (watering treatment x temperature) testing the hypothesis that the regression lines had the same slope, or the effect of watering treatment was not dependent on tree canopy temperature. If the interaction was not significant it was removed and the slopes of each regression line were assumed equal to test the hypothesis that each line’s y-intercept was the same, or that there was no effect of watering treatment on the response.

The residuals of temperature and *M*. *tenebricosa* abundance for 2014 and 2015 did not meet the assumptions of normality. Since log transforming count data to meet parametric assumptions can produce models with poor fits compared to raw data fit to a Poisson distribution, we compared the goodness of fit for GLM models using raw and log-transformed data [41]. Model comparisons for the analyses of temperature and water treatment predicting untransformed *M*. *tenebricosa* abundance from 2014 and 2015 were conducted using PROC GENMOD in SAS 9.4 (SAS Version 9.4, Cary, NC), specifying a Poisson distribution and log link function. Next, we analyzed log10-transformed *M*. *tenebricosa* abundance from each year using PROC GENMOD, specifying a normal distribution and log link function. Based on goodness of fit indices (AIC and Deviance/DF), we used log10-transformed *M*. *tenebricosa* abundance for both analyses (S1 Table).

## Results

### Effect of temperature and water treatment on drought stress

**Fig 2:**
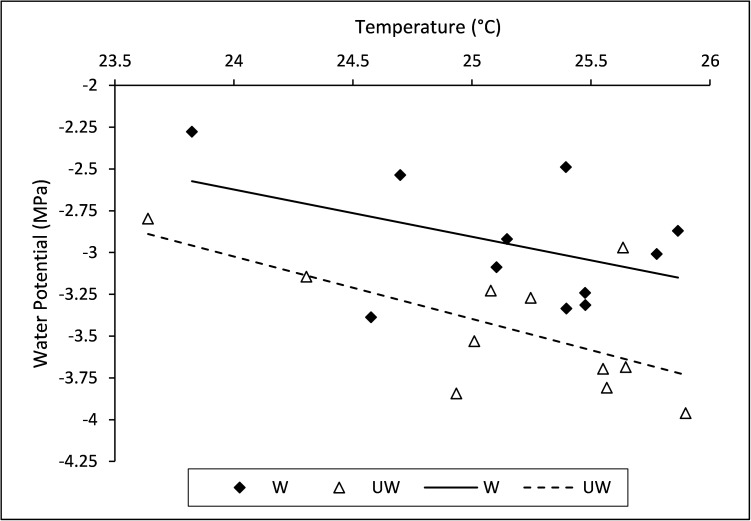
ANCOVA regression plot of tree canopy temperature and watering treatment (W = watered, UW = unwatered) predicting mean xylem water potential (MPa).

Mean tree canopy temperature from May 2014 through August 2014 ranged from 23.64 to 25.90°C with a mean (±SEM) of 25.15 (±0.13). Water potential of the watered trees ranged from -2.28 to -3.39 MPa with a mean (±SEM) of -2.95 (±0.11) and unwatered trees ranged from -2.80 to -3.96 MPa with a mean (±SEM) of -3.45 (±0.12). There was no interaction between temperature and water treatment. The full model of temperature and watering treatment predicting xylem water potential was significant (Table 1). As temperature increased, *A*. *rubrum* xylem water potential (MPa) decreased and was, on average, more negative on unwatered than watered trees (Table 1, Fig 2).

**Table 1:** ANCOVA of the effect of temperature and water treatment on xylem water potential.

Variable	Type III SS	Error Df	*F*	*P*
Water Potential	4.28	21	10.49	**0.0009**
Temperature	0.88	21	8.26	**0.0097**
Water Treatment	1.39	21	13.06	**0.0019**

Interaction term was not significant. Statistics represent model without an interaction term. Significant p-values are in bold.

Xylem vulnerability curves conducted by Johnson et al. [42] suggest that *A*. *rubrum* is moderately drought tolerant and can withstand as low as -3.9 MPa of xylem negative pressure before embolism occurs in 50% of vessels, indicative of severe drought stress. We found that the warmest, unwatered *A*. *rubrum* street trees surpassed -3.9 MPa during midday water potential measurements (Fig 2), suggesting that damaging levels of drought stress are occurring in Raleigh’s *A*. *rubrum* street trees that do not receive irrigation.

### Effect of temperature and water treatment on *M*. *tenebricosa* fitness and abundance

**Fig 3:**
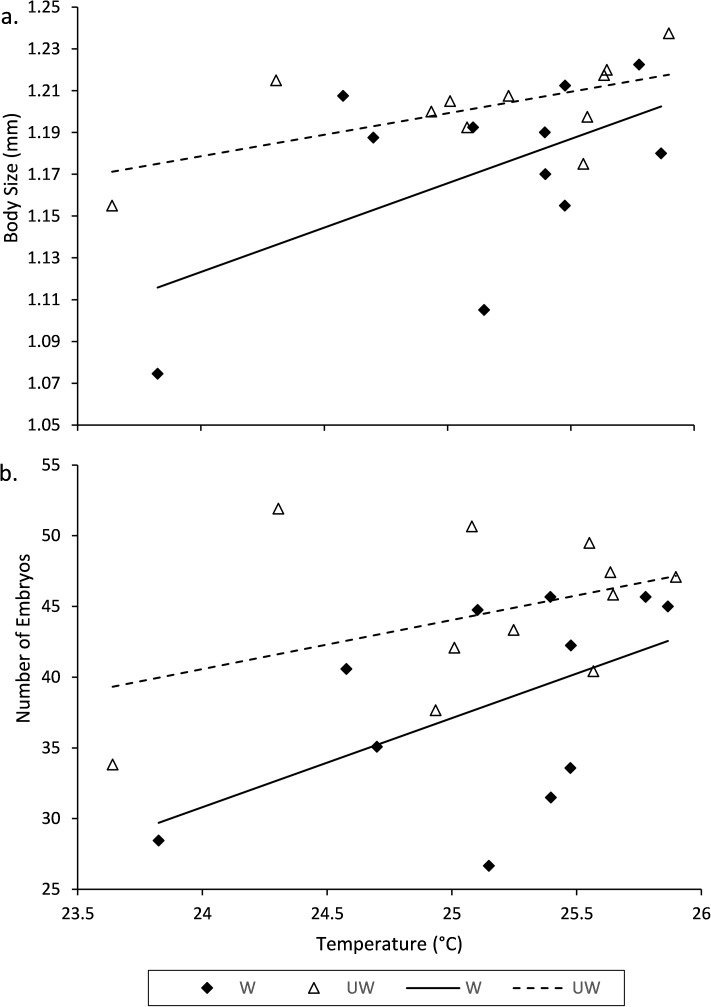
ANCOVA regression plots of (a) tree canopy temperature predicting mean *M*. *tenebricosa* adult female body size of individuals from each watering treatment (W = watered, UW = unwatered) and (b) tree canopy temperature predicting mean number of embryos per individual *M*. *tenebricosa* adult female on each watering treatment.

The effect of temperature on adult female body size did not depend on drought stress. Adult female *M*. *tenebricosa* body size was significantly associated with the full model of tree canopy temperature and drought stress (Fig 3A). Body size increased with tree canopy temperature (Table 2), corroborating Dale and Frank [5]. More importantly, *M*. *tenebricosa* on unwatered trees were 3% larger than those on watered trees (Table 2).

**Table 2:** ANCOVA of the effect of temperature and water treatment on *M*. *tenebricosa*.

Response	Predictor	Type III SS	Error Df	*F*	*P*
Body size		0.03	19	6.21	**0.0084**
	Temperature	0.007	19	7.48	**0.0131**
	Water treatment	0.005	19	5.14	**0.0353**
Embryo production		1072.09	19	5.74	**0.0112**
	Temperature	177.54	19	5.05	**0.0367**
	Water treatment	232.99	19	6.62	**0.0186**
Log10(x+1) Initial abundance		15.90	19	6.67	**0.0064**
	Temperature	6.46	19	13.14	**0.0018**
	Water treatment	0.13	19	0.27	0.6092
Log10(x+1) Final abundance		17.65	17	7.23	**0.0053**
	Temperature	7.53	17	13.43	**0.0019**
	Water treatment	0.58	17	1.04	0.3216
Log Population growth ratio		0.25	17	0.35	0.7079
	Temperature	0.16	17	0.61	0.4446
	Water treatment	0.08	17	0.09	0.7644

No interaction terms were significant. Statistics represent models without an interaction term. Significant p-values are in bold.

The effect of temperature on adult female embryo production was also not dependent on drought stress. *Melanaspis tenebricosa* embryo count was associated with the full model of temperature and water treatment and adult females produced significantly more offspring as tree canopy temperature increased (Fig 3B, Table 2). More strikingly, *M*. *tenebricosa* on unwatered trees produced 17% more embryos per individual than those on watered trees (Table 2). Thus, *M*. *tenebricosa* on the warmest unwatered trees have the potential to produce substantially more successful offspring than those on the coolest watered trees.

**Fig 4:**
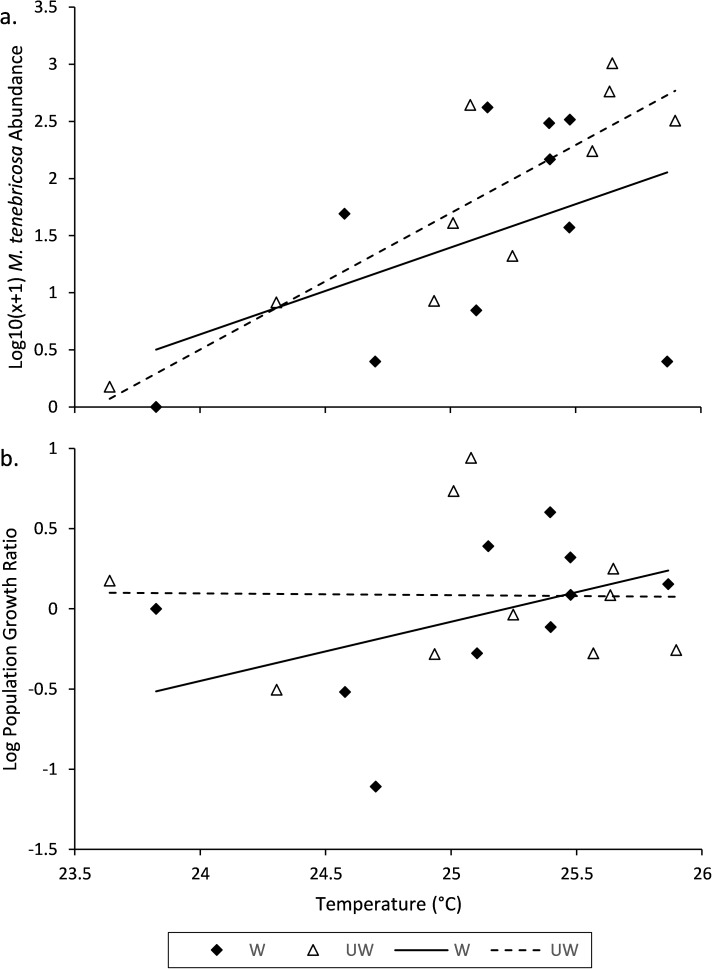
ANCOVA regression plot of (a) tree canopy temperature predicting final *M*. *tenebricosa* abundance (2015) on study trees (W = watered, UW = unwatered) and (b) tree canopy temperature predicting the *M*. *tenebricosa* log10 population growth ratio of individuals on watered and unwatered trees.

*Melanaspis tenebricosa* abundance in April 2014 ranged from 0 to 577 live adult females per 0.3 m of twig with a mean (±SEM) of 160.73 (±41.61). *M*. *tenebricosa* abundance in September 2015 ranged from 0 to 1018 live adult females per 0.3 m of twig with a mean (±SEM) of 194.39 (±59.32). At the beginning of the experiment (April 2014), tree canopy temperature and *M*. *tenebricosa* abundance were significantly, positively associated, but did not differ between treatments (Table 2). By the conclusion (September 2015), tree canopy temperature remained significantly associated with *M*. *tenebricosa* abundance, but there was still no difference in abundance between watering treatments (Table 2, Fig 4A). Population growth ratios were also not significantly predicted by tree canopy temperature and did not differ between watering treatments (Table 2, Fig 4B).

## Discussion

The rate and severity of global climate change and extent of urbanization are increasing, yet we still have inconsistent predictions about how heat and drought will affect herbivorous insects [43–45]. Inconsistencies are partly the result of single-factor and observational studies, which ignore the synergistic and additive relationships that affect ecological processes [46]. In fact, few studies have simultaneously examined the effect of temperature and drought on herbivorous pests, and none have manipulated drought on established urban trees [24, 45, 47]. Here we show that drought exacerbates the effect of warming such that *M*. *tenebricosa* produced over 17% more embryos on the warmest unwatered trees than the warmest watered trees, and over 65% more than the coolest watered trees. Understanding how multiple climatic factors like temperature and drought act together to affect insect pests and their host trees is essential for managing forests and ecosystem services under climate change [25, 48].

There is mixed support in the literature about the effects of plant drought stress on herbivorous arthropods, but sap-feeding insect performance generally increases with plant stress [14, 45, 47, 49]. On trees in particular, drought stress increases sugar and nitrogen concentrations, which herbivores are able to exploit [16, 47]. Since herbivorous insects are primarily nitrogen-limited, greater plant nitrogen concentrations can increase fecundity, development rate, and abundance [18, 47, 50]. For example, McClure [51] showed that the fecundity, survival, and abundance of an armored scale insect, *Fiorinia externa*, increased as percent total nitrogen content of eastern hemlock trees increased. Although we don’t know how nutrient content differed among our study trees, our results suggest that tree drought stress benefits pest fitness, and nutrient content is a plausible mechanism for this.

Characteristics of urban forests, like the range of warming found within a city, are often similar to those projected in natural forests by the end of this century [44]. Research has demonstrated that insects and plants respond to warming in urban and natural forests [38, 52]. However, these forests face multiple factors that may interact or have additive effects on the services they provide [25]. The net effects of multifactor interactions are difficult to capture, but recent work is beginning to do so. For example, Meineke et al [53] found that spider mite and scale insect pests were more abundant on warmer than cooler urban trees, yet warmer temperatures, not pests, reduced carbon sequestration by 12% across an urban forest. Our results suggest that temperature and drought additively affect an important insect pest of the most common genus of urban tree in eastern North America [31]. Evidence from Dale & Frank [40] suggests that increased temperatures, *M*. *tenebricosa* abundance, and drought stress reduce the condition and services provided by these urban trees. Thus, our results are important because they can inform urban forest management practices like irrigation and landscape design, or by guiding tree species selections based on urban landscape characteristics, which may mitigate the negative effects of cities [54, 55].

An important note about the effect of drought and temperature on *M*. *tenebricosa* and similar insect pests is the extended timeline by which change occurs. On our study trees, *M*. *tenebricosa* abundance increased by three orders of magnitude across just over a 2°C increase in tree canopy temperature (Fig 4A). However, this occurred over many years of infestation and exposure to the urban environment. Just one year of watering alleviated drought stress and resulted in smaller insects with lower embryo production compared to individuals on drought stressed trees. Although our population growth and abundance results do not suggest that watering reduced insect abundance, this may be due to our limited ability to detect a change. More specifically, the slow rate at which these insects reproduce, the large initial population size infesting our study trees, and the variation in scale abundance among trees makes detection difficult. Therefore, more long-term evaluation of the effect of reducing drought stress is needed. This also emphasizes the importance of reducing water stress throughout the ontogeny of *A*. *rubrum* street trees, particularly early on, so that *M*. *tenebricosa* cannot grow to damaging populations before mitigation efforts begin.

Urban warming, drought stress, and *M*. *tenebricosa* abundance reduce *A*. *rubrum* tree condition and ecosystem services by reducing canopy density, photosynthesis rates, and aesthetic quality [39, 40]. Therefore, chronic pest infestations of *M*. *tenebricosa* and other scale insects pose an inconspicuous, yet serious threat to urban forests as cities expand and climatic conditions become warmer and drier [40, 56]. Although understudied, evidence suggests that chronic feeding by herbivores like scale insects or aphids is widespread and increasing due to global warming [57, 58]. Since some abiotic conditions of urban forests, particularly temperature and drought, often resemble those projected in natural forests in multiple regions of the world, our results could inform future changes in pest dynamics in other systems and natural forests [44, 59–61]. Similarly, cities may experience impending pest outbreaks associated with climate change before surrounding natural forests [62]. Future studies should take an analogous approach by manipulating multiple urban environmental stress factors and measuring their effect on insects and plants under field conditions. Only then may we obtain a more complete understanding of the effects of future global change on urban and natural ecosystems.

## Supporting information

S1 Table ANCOVA model comparisons of temperature predicting *M*. *tenebricosa* abundance. (DOCX)
