# Supplementary material for: Warming and drought combine to increase pest insect fitness on urban trees
Source: PLoS One. 2017 Mar 9;12(3):e0173844. doi: 10.1371/journal.pone.0173844 (PMC5344462; doi:10.1371/journal.pone.0173844)
Supplement: S1 Table — (DOCX) [file pone.0173844.s001.docx]

| **Dependent variable** | **Transformation** | **Model Distribution** | **AIC** | **Deviance/DF** |
| --- | --- | --- | --- | --- |
| 2014 *M. tenebricosa* abundance | None | Poisson | 2449.10 | 122.21 |
|  | Log10(x+1) | Normal | 52.79 | 0.52 |
| 2015 *M. tenebricosa* abundance | None | Poisson | 3924.68 | 224.13 |
|  | Log10(x+1) | Normal | 51.68 | 0.61 |

**S1 Table. ANCOVA model comparisons of temperature predicting *M. tenebricosa* abundance**
